# The Dynamics of T and B Cells in Lymph Node during Chronic HIV Infection: TFH and HIV, Unhappy Dance Partners?

**DOI:** 10.3389/fimmu.2016.00522

**Published:** 2016-11-22

**Authors:** Jung Joo Hong, Kyu-Tae Chang, Francois Villinger

**Affiliations:** ^1^National Primate Research Center (NPRC), Korea Research Institute of Bioscience and Biotechnology (KRIBB), Cheongju, South Korea; ^2^New Iberia Research Center, University of Louisiana Lafayette, Lafayette, LA, USA

**Keywords:** TFH cells, hyperplastic follicle, germinal center, HIV, SIV

## Abstract

Although the dynamics of germinal center (GC) formation, follicular helper T (TFH) cell recruitment to *B cell follicle*s within lymphoid organs, and changes of lymphoid tissue architecture in HIV/SIV infection have been documented, the underlying immunopathology remains unclear. Here, we summarize what is known regarding the kinetics of TFH cells and *GC B cells* during the course of infection as well as the potential immunopathological features associated with structural changes in the lymphoid compartment. This review also explores the implications of cell dynamics in the formation and maintenance of viral reservoirs in hyperplastic follicles of secondary lymphoid organs before and after viral suppressive antiretroviral therapy.

## Introduction

The ongoing human immunodeficiency virus (HIV) pandemic continues unabated with over 37 million people infected in spite of the availability of a large number of antiretroviral drugs (1). The current combination antiretroviral therapy (ART) while highly effective at controlling viral replication, is however unable to eliminate the virus, which readily rebounds upon ART cessation. Therefore, the development of a protective vaccine remains a priority, though this task is complicated by a relatively poor understanding of immune correlates of protection at this time. In addition, the persistence of HIV infection in spite of potent combinations of drugs also remains to be fully elucidated. Even more puzzling, the ongoing vigorous *but inadequate* antiviral immune response both during and post ART remains unable to contain chronic virus replication. Most active HIV replication occurs in CD4 T cells in secondary lymphoid organs (2, 3), and recent data also highlights these sites as important reservoirs of latent infection during ART (4, 5). Moreover, these reservoirs are seeded early postinfection (6), *and early ART may decrease the size of cells harboring HIV DNA (*7, 8*), although an exact temporal relation between seeding magnitude of various anatomical reservoirs and specific cell lineages remains to be fully elucidated for both HIV and SIV*. During the course of infection, virus has been shown to remain in the germinal center (GC) of hyperplastic follicles, while the architecture of the lymph node experiences gradual remodeling (2, 9).

## Generation of Follicular Hyperplasia/Involution

Persistent immune activation is a hallmark of chronic HIV/SIV infections, which induces a progressive pathology not only in peripheral blood but also in secondary lymphoid tissues, causing a profound remodeling of the lymph node architecture throughout the course of infection. Histological patterns of structural alteration of lymphoid architecture were already well described in the 1980s, in the context of HIV infection (10–13). Specifically, lymph node morphology in HIV patients with or without AIDS is characterized initially by follicular hyperplasia, followed by involution, resulting in the lymphadenopathy and destruction of follicular architecture, which helped in the original diagnosis of AIDS (14). In SIV and SHIV infection of non-human primate models of human HIV infection, rhesus macaques that followed an accelerated disease course and died within 6 months, severe follicular involution was observed in their lymphoid tissues, while on the contrary, animals that survived longer had lymphadenopathy with confluent GCs and follicular hyperplasia (15, 16). Histologic and cellular characterization of lymph nodes during infection revealed similar structural alterations among SIV-infected rhesus macaques and HIV-infected patients. Although the mechanisms are not fully elucidated yet, gradual histological alterations such as the deposition of collagen and non-amyloid substance may be associated with follicular involution at the end stage of HIV infection (17, 18). Exposure of follicular dendritic cells to HIV may create an inflammatory environment and lead to the impaired survival of follicular B cells (19). The magnitude of GC reactions in follicular hyperplasia or involution, if any, is closely linked to the regulation of follicular helper T (TFH) cells.

## Follicular Helper T Cells in the GCs of Hyperplastic Follicles During the Course of HIV Infection

**Figure 1:**
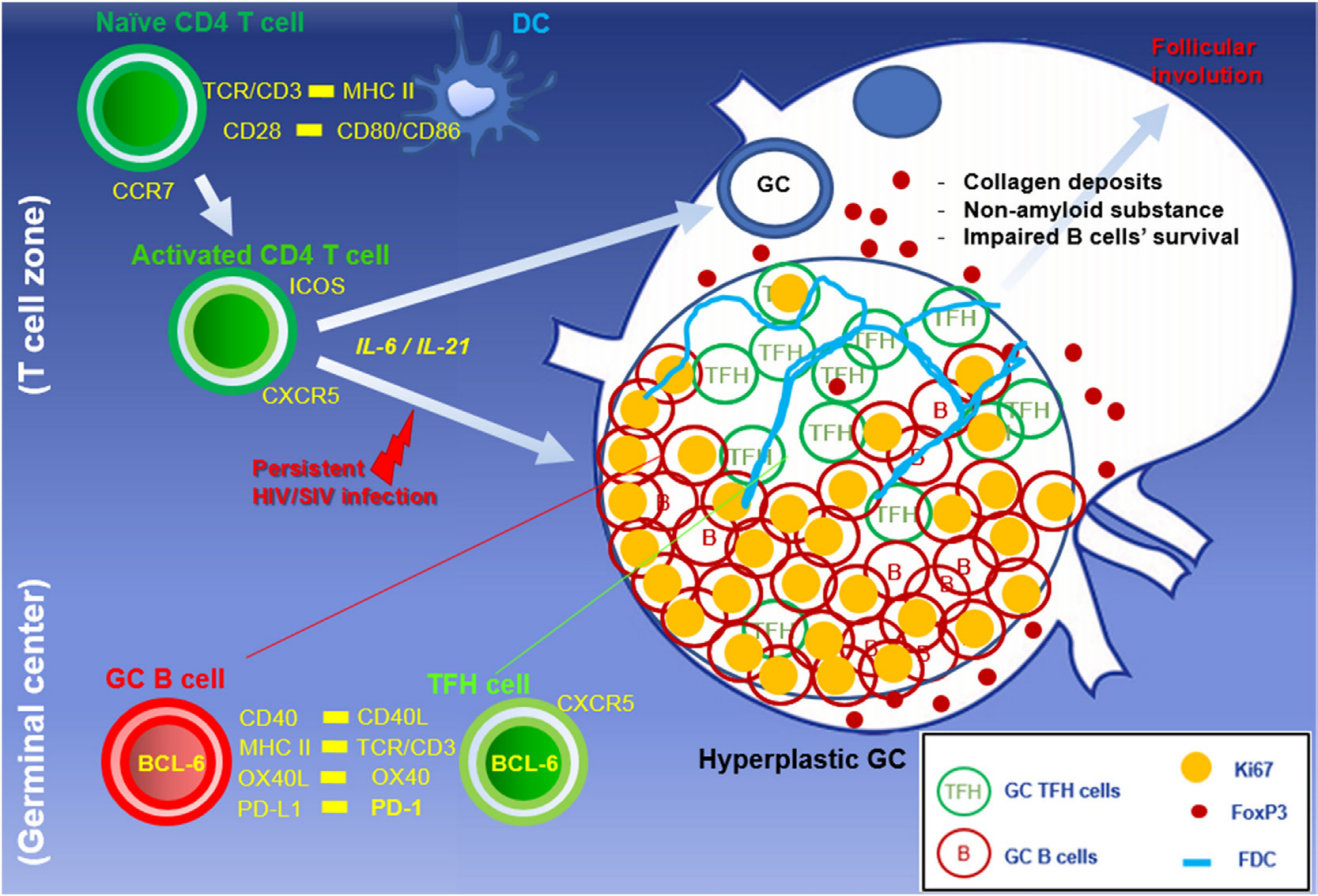
**Abnormal accumulation of TFH cells in hyperplastic GC during HIV/SIV primary infection**. Naïve mature CD4 T cells are activated through dendritic cells. The persistent viral antigens stimulate primed CD4 T cells, resulting in the formation of hyperplastic GC with the massive B cell expansion, TFH accumulation, and development of network of follicular dendritic cells. Treg and PD-L1 expressing cells within GCs are capable of modulating GC TFH cells to suppress GC-related responses at the end stage of HIV infection.

Specific CD4 T helper cells termed TFH cells differentiate from precursors under the control of the transcription factor Bcl6 and are characterized by their function, which is to provide T cell help for B cells, and are distinct from other CD4 T cell subsets such as Th1, Th2, and Th17 cells (20). In lymphoid tissues, information on the location of CD4 T cells within follicles is also vital to identifying resident TFH cells (Figure 1). Although there is no single marker for distinguishing TFH cells from other CD4 subsets, they are defined by their expression of surface co-stimulatory molecules CXCR5, CD200, ICOS, and a high density of PD-1 (20, 21). These memory type cells generally express low levels of CCR7 but are able to migrate toward B cell follicles in lymphoid organs, produce IL-21, and deliver B cell help in the GC environment for the development of T cell-dependent humoral adaptive immunity (22, 23). Studies using knock-out mice for IL-6 and IL-21 have shown that both are necessary for TFH differentiation and GC development in secondary follicles. IL-6-deficient mice exhibited a marked defect in GCs formation, STAT1 and STAT3 signaling, downstream IL-21 production, and IgG production primarily due to the lack of TFH differentiation *in vivo* (24). IL-21 deficiency also results in a severe reduction of GCs though this signal seems downstream of the one caused by IL-6 deficiency (25). Moreover, IL-6 and IL-21 appear to regulate the generation of TFH cells in the absence of Th1, Th2, and Th17 cells (26), suggesting that conversely, increases in the expression of IL-6, IL-21, or both cytokines may lead to lymphoid hyperplasia and rapid development of GCs, as observed during HIV and SIV infection. In fact, in HIV-infected individuals, a decrease in levels of circulating IL-21 and decreased production of IL-21 by CD4 cells was noted in blood (27). Similar to HIV infection, substantial depletion of IL-21^+^ CD4 T cells was reported in the blood in SIV-infected macaques (28). In lymphoid tissues of HIV patients, however, a marked expansion of IL-21-secreting TFHs was noted (29). Moreover, concurrent accumulation of TFH cells and particularly within the GCs of lymph node follicles and more precisely at the periphery of the GC had significantly increased IL-21 expression during SIV infection (30), suggesting trafficking of IL-21-producing TFH cells during the chronic immune activation characteristic of chronic SIV infection.

**Figure 2:**
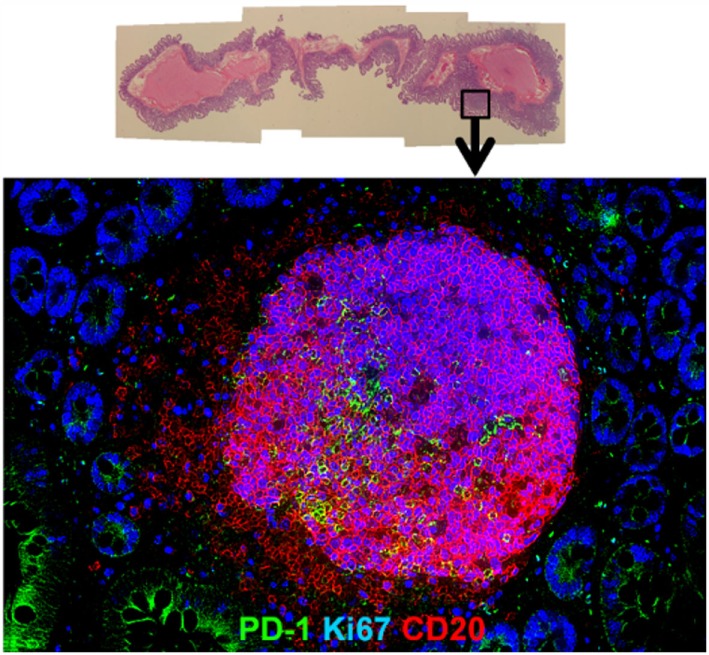
**Hyperplastic follicle in gut tissue during chronic SIV infection**. Representative H&E (upper) and immunofluorescence image (lower) of hyperplastic follicle staining with Ki67 (blue), PD-1 (green), and CD20 (red) in ileum from a chronically SIV-infected rhesus macaque.

Upon HIV infection, there is a rapid infiltration of these TFH cells and formation of numerous GCs within lymphoid organs, characteristic of lymphocyte hyperplasia seen early in chronic infection. Recent studies demonstrated that HIV-infected patients displayed an aberrant accumulation of TFH cells compared to uninfected individuals (29). Similar observations were reported in lymph nodes, spleen, and gut tissues of rhesus macaques, in which the resident TFH cells (PD-1^high^ CD4^+^ T cells) within GCs of hyperplastic follicles were markedly expanded, with a parallel increase and accumulation of Ki67^+^ GC B cells during chronic SIV infection (31, 32) (Figure 2). Of interest though, was the observation that as TFH accumulated within GCs, their expression of Ki67 decreased with up to 80% of TFH negative for this proliferation marker, suggesting that the continued input of this lineage to be contributed from cells migrating into follicles rather than local proliferation (30), and potentially, these cells have reached a terminal differentiation stage and function, which is to deliver help to local B cell differentiation and maturation. These findings are consistent with the limited proliferative capacity of human TFH cells whereby cross-linking their high level of PD-1 may dissociate continuous TCR signaling.

Understanding whether GC TFH cells accumulated during HIV/SIV infection are viral antigen-specific is also important. However, this has, hitherto, rarely been addressed because of the difficulty in identifying their responses. In this respect, there is also little experimental evidence demonstrating the dynamics between antigen-specific TFH cells and hyperplastic GCs. Interestingly, two recent articles have reported a novel assay to determine the frequencies of antigen-specific TFH cells within secondary lymphoid tissues of humans and macaques using cytokine-independent activation-induced markers CD25 and OX40 (33, 34). Such new technique is expected to markedly enhance our comprehension of the role of antigen specificity in the lymphoid hyperplasia that is observed during SIV/HIV infection.

## Negative Regulation of TFH Cells in Hyperplastic Follicle

**Figure 3:**
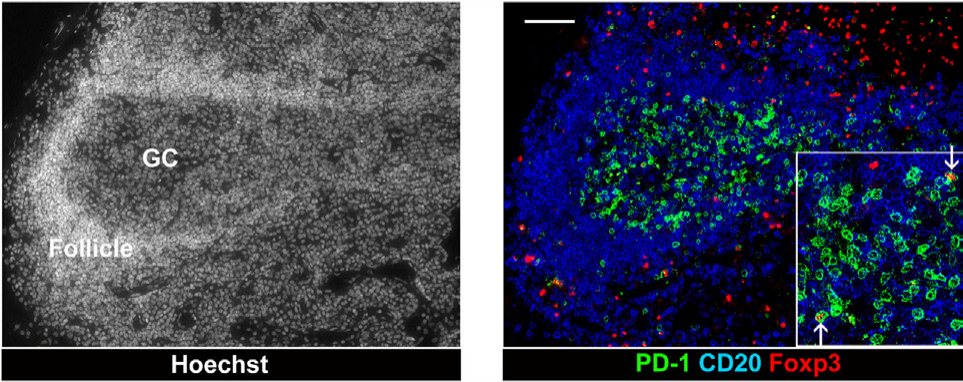
**Follicular T regulatory cells in a hyperplastic follicle *of lymph node from chronic SIV infection***. Foxp3^+^ cells and PD-1 high cells in an expanded GC of a hyperplastic follicle. The lymph node biopsies were stained with CD20 (blue), PD-1 (green), Foxp3 (red), and Hoechst dye (white). Scale bars = 50 μm.

Unlike GC B cells, the frequency of proliferating GC TFH cells drops once hyperplastic follicles are established during infection. There are several potential negative regulators able to suppress resident TFH cells from the persistent division in the local environment. First, a series of recent findings suggest that Foxp3^+^ regulatory T (Treg) cells also arise in the lymphoid compartment and may play an important role in the downregulation of TFH cell-mediated GC development. In mouse and human studies, a subset of TFH cell with a surface profile of Treg cells has been detected within GCs, which negatively regulated TFH cell-dependent B cell responses *in vitro* (35–37). So far, monitoring follicular Treg cells during follicular hyperplasia by HIV has been described, but few studies have focused on this issue. In both HIV and SIV infections, the density of Treg cells increase in the T cell zone but not in follicular area (38) (Figure 3). Petrovas et al. defined Foxp3^+^ cells among TFH cells in a SIV model and reported no expansion during the course of infection (32). *In situ* analyses using immunofluorescent staining for Foxp3, PD-1, CD20, and nuclei revealed that Foxp3^+^ cells are more abundant outside than inside follicles, and only few FoxP3^+^ PD-1^high^ cells were present within GCs of hyperplastic follicles in SIV-infected macaque (Figure 1). Indeed, the frequency of Treg cells among TFH cells is decreased in chronic SIV infection (39). The abundance of IL-21 may be associated with the suppression of Treg cells in select chronic inflammatory situations (40).

Another possible mechanism of down-modulation of TFH cells is signaling through PD-1, leading to decreased T cell proliferation; TFH cells are likely very sensitive to this mechanism because of their high-level expression of PD-1. In the setting of chronic HIV infection, PD-1 ligands on antigen-presenting cells including B cells were upregulated in both blood and lymph nodes (41–43), leading to functional impairment of PD-1 expressing T cells. Macaque studies have shown that PD-L1 expression was increased on dendritic cell populations in blood and lymph node post SIV infection, compared to uninfected controls (44). Of note, DC-like cells expressing PD-L1 were markedly increased locally with exaggerated GC formation, and they eventually interact with TFH cells in hyperplastic follicles during chronic SIV infection (31). PD-1/PD-L1 interaction was shown to induce a decrease in the proliferation, survival, and cytokines secretion of human TFH cells (43). These findings suggest that the suppression of proliferation of resident TFH cells may be more readily associated with the cross-linking of PD-1 with PD-L1 in hyperplastic follicles than the presence of Foxp3^+^ Treg cells during HIV infection.

Third, Chronic immune activation of lymphoid tissues leads to a gradual remodeling of the architecture, resulting in follicular involution. In HIV patients, collagen was gradually deposited into T cell zone of lymphoid node (45). This fibrosis inversely correlates with the presence of naïve CD4 T cells (46). TFGβ activated by inflammation induces collagen deposition, resulting in the loss of the fibroblastic reticular cells (FRCs) network that is associated with the production of growth factors such as IL-7. This loss leads to the death of T cells and then decrease in lymphotoxin-β essential for the survival of FRCs (47), suggesting the disruption of cell to cell contact between FDCs, TFH, and B cells.

## TFH Serve as a Long-Lived Viral Reservoir

Resident TFH appear to not express CCR5 (48, 49), yet many are infected and replicate HIV-1 (50) and SIV (51). This leads to the question of site of infection: are they being infected before migrating into the GC, infected by cell–cell transmission from FDCs in GCs, or is there enough co-receptor expression to allow for infection even though CCR5 levels are too low to be detected by flow or *in situ* techniques? This aspect of TFH infection will require additional work to be resolved, though what has become readily apparent is that hyperplastic follicles with high density of resident TFH cells can serve as the latent virus reservoir during the course of infection even in elite controllers (51). Thus, the extent of infection in TFH of hyperplastic follicles in lymph nodes needs to be taken into account in any strategy aimed at reducing or eliminating latent viral reservoirs. Moreover, the analyses of antiretroviral drug penetration and conversion to their active form have only begun to be examined. Recent data suggest that treatment with ART appears to decrease the relative frequency of GC TFH cells in lymph nodes of HIV patients, perhaps secondary to partial resolution of the immune activation, though the relative frequency is still higher than in uninfected individuals (29, 52), *and evidence of lower concentrations of ART in lymphatic tissues and relative to peripheral blood have been reported (*53*) including data showing lower conversion within GCs relative to the other lymphoid areas (personal communication)*. Although HIV RNA is rarely detected in the GCs of lymph node during antiretroviral therapy, viral proteins such as Gag p24 and HIV DNA remain detectable over a substantial period time (54, 55). Blood, Lymph node, and splenic TFH cells show higher level of SIV RNA compared with non-TFH cells in the presence of combined ART (51, 56). Other lymphoid tissues such as gut possess latently infected cells despite undetectable plasma HIV RNA in patients with long-term ART treatment (57), which would be conducive to reseed and rekindle of infection in blood and other secondary lymphoid organs. Importantly, hyperplastic follicles still exist in HIV patients’ after extended ART (58). It is quite possible that GC TFH cells accumulated may reactivate virus replication upon ART cessation, serving as a major source of virus rebound. Overall, the existence of hyperplastic GCs may represent an impediment to a cure for HIV-1 infection and must expressly be addressed in HIV-1 therapeutic strategies.

## Conclusion

In conclusion, TFH cells not only are a critical component of the immune response but also serve an active and latent reservoir for HIV/SIV infection. A better understanding of TFH cell kinetics and their role as a latent cell reservoir is clearly needed for any ART and/or immune-based interventions to control virus replication in the absence of ART. Therefore, the recent efforts at an understanding of TFH-related GC immune responses during HIV disease will, in spite of much difficulty, likely provide major advances in the generation of therapeutic strategies to target the potential latent reservoirs of HIV and ultimately its eradication.

## Author Contributions

All authors listed have made substantial, direct, and intellectual contribution to the work and approved it for publication.

## Conflict of Interest Statement

The authors declare that the research was conducted in the absence of any commercial or financial relationships that could be construed as a potential conflict of interest.
